# Secreted phospholipase A_2_-IIA-induced a phenotype of activated microglia in BV-2 cells requires epidermal growth factor receptor transactivation and proHB-EGF shedding

**DOI:** 10.1186/1742-2094-9-154

**Published:** 2012-07-02

**Authors:** Rubén Martín, Claudia Cordova, Maria L Nieto

**Affiliations:** 1 Instituto de Biología y Genetica Molecular (IBGM), CSIC-UVa, Valladolid, Spain; 2 ICICOR, Hospital Clínico, Valladolid, Spain

**Keywords:** Microglia, Secreted phospholipase A_2_-IIA, Proliferation, Phagocytosis, Epidermal growth factor receptor

## Abstract

**Background:**

Activation of microglia, the primary component of the innate immune response in the brain, is a hallmark of neuroinflammation in neurodegenerative disorders, including Alzheimer’s disease (AD) and other pathological conditions such as stroke or CNS infection. In response to a variety of insults, microglial cells produce high levels of inflammatory cytokines that are often involved in neuronal injury, and play an important role in the recognition, engulfment, and clearance of apoptotic cells and/or invading microbes. Secreted phospholipase A_2_-IIA (sPLA_2_-IIA), an enzyme that interacts with cells involved in the systemic immune/inflammatory response, has been found up-regulated in the cerebrospinal fluid and brain of AD patients. However, despite several approaches, its functions in mediating CNS inflammation remain unknown. In the present study, the role of sPLA_2_-IIA was examined by investigating its direct effects on microglial cells.

**Methods:**

Primary and immortalized microglial cells were stimulated by sPLA_2_-IIA in order to characterize the cytokine-like actions of the phospholipase. The hallmarks of activated microglia analyzed include: mitogenic response, phagocytic capabilities and induction of inflammatory mediators. In addition, we studied several of the potential molecular mechanisms involved in those events.

**Results:**

The direct exposure of microglial cells to sPLA_2_-IIA stimulated, in a time- and dose-dependent manner, their phagocytic and proliferative capabilities. sPLA_2_-IIA also triggered the synthesis of the inflammatory proteins COX-2 and TNFα. In addition, EGFR phosphorylation and shedding of the membrane-anchored heparin-binding EGF-like growth factor (pro-HB-EGF) ectodomain, as well as a rapid activation/phosphorylation of the classical survival proteins ERK, P70S6K and rS6 were induced upon sPLA_2_-IIA treatment. We further demonstrated that the presence of an EGFR inhibitor (AG1478), a matrix metalloproteinase inhibitor (GM6001), an ADAM inhibitor (TAPI-1), and a HB-EGF neutralizing antibody abrogated the phenotype of activated microglia induced by the sPLA_2_-IIA.

**Conclusion:**

These results support the hypothesis that sPLA_2_-IIA may act as a potent modulator of microglial functions through its ability to induce EGFR transactivation and HB-EGF release. Accordingly, pharmacological modulation of EGFR might be a useful tool for treating neuroinflammatory diseases characterized by sPLA_2_-IIA accumulation.

## Background

Microglial cells are considered as central nervous system (CNS)-resident professional macrophages. They constantly survey the brain parenchyma and react immediately to changes in the microenvironment, becoming readily activated in response to infection or injury [1]. They may play a dual role, participating in host defense of the brain and tissue repair, as well as acting as phagocytes to engulf tissue debris and dead cells. However, microglia can also contribute to the establishment or exacerbation of tissue damage depending on the type or intensity of the harmful stimulus.

Cerebral ischemia and other neurodegenerative disorders such as Alzheimer's disease (AD), Parkinson’s disease, and multiple sclerosis, among others, are associated with proliferation and activation of microglia [2-6]. The activated microglia undergo dramatic morphological changes, from a resting ramified form to an activated amoeboid shape, and secrete a host of immunomodulatory and neurotoxic factors. Whilst significant advances have been made to identify the contribution of the cytotoxic agents released from microglia to the neurodegenerative process, it is less clear and remains to be determined which factors trigger microglial activation in these various disorders.

In neurodegenerative diseases such as Alzheimer’s, for example, players involved in the inflammatory process include S100a9, β-amyloid peptides (Aβ), macrophage colony-stimulating factor and acute-phase proteins such as C-reactive protein, amyloid P and secreted phospholipase A_2_-IIA (sPLA_2_-IIA), among others [2,7-12]. Recent studies have revealed that S100a9, Aβ and macrophage colony-stimulating factor themselves can promote the reactivity of microglia to enhance their neurotoxicity. However, any role that sPLA_2_-IIA might play in microglia activation is still unknown.

Secreted phospholipases A_2_ represent a family of eleven low molecular mass, calcium- dependent lipolytic enzymes. They catalyze the hydrolysis of the sn-2 ester bond of glycerophospholipids present in cell membranes to form essential cell-signaling molecules. They are widely distributed in human tissues including brain, where their specific function is still largely unclear [13,14], although current evidence suggests that sPLA_2_s may affect some neuronal functions, such as neuritogenesis, neurotoxicity, neurotransmitter release and survival [15-20]. Different levels of sPLA_2_ activity have been found in various regions of the central nervous system in both humans and rodents, and the subtypes identified include sPLA_2_-IIA, IIC, IIE, II, V, X and XII.

Secreted PLA_2_-IIA (sPLA_2_-IIA) was first identified in synovial fluid, and then characterized as an acute-phase protein under the transcriptional control of pro-inflammatory cytokine signaling [21]. Later on, its presence in tears was reported and it came to be considered a powerful innate ocular surface barrier against Gram-positive bacteria [22]. Its serum levels dramatically increase under pathological conditions that involve systemic inflammatory processes such as sepsis, rheumatoid arthritis, and cardiovascular disease (up to 1000-fold and >1 μg/ml). Additionally, enhanced expression of sPLA_2_-IIA has also been found in certain neurological disorders and as a result of brain insult, it being associated with CNS injuries such as cerebral ischemia or mechanical damage to spinal cord tissue [18,23-26]. Recent reports have shown it to be up-regulated in both cerebrospinal fluid and brain of patients with Alzheimer’s disease. In fact, increased immunoreactivity for sPLA_2_-IIA has been reported in reactive astrocytes of the cortex and hippocampus (restricted mainly to the dentate gyrus and CA3 field) around Aβ-containing plaques [11,12].

sPLA_2_-IIA, as well as other sPLA_2_ subtypes, can also exert various biological functions and transduce signals independently of their catalytic activity through receptors or binding proteins such as M-type receptor, factor Xa, integrin αvβ3 and α4β1, heparan sulfate and proteoglycans, etcetera [27-31]. Indeed, it has been reported that sPLA_2_-IIA influences survival of some cellular types within the CNS including oligodendrocytes and neurons, independently of its catalytic activity [19,24].

In this study, we provide data demonstrating the functional consequences of microglial cell exposure to the activating agent sPLA_2_-IIA. We have measured proliferative responses, phagocytic capabilities and synthesis and release of several molecules with pro-inflammatory activities, for example, tumor necrosis factor-α (TNFα) and cycloxigenase-2 (COX-2), as indices of microglial activation. In addition, we have characterized several of the potential molecular mechanisms involved in these events.

## Methods

### Reagents

A C127 mouse fibroblast cell line, stably transfected with the coding sequence of sPLA_2_-IIA from human placenta, was kindly provided by Dr Olivier (Nancy University Hospital, Nancy Cedex, France) and used as a source of human recombinant enzyme (hr-sPLA_2_-IIA) in some experiments to ascertain specificity [32]. sPLA_2_-IIA was obtained and purified as described previously [33]. The absence of lipopolysaccharide (LPS) in the preparation was confirmed by the limulus amebocyte lysate assay test in the batches used for the experiments. Moreover, experiments are conducted in the absence of fetal calf serum (FCS), which ensures that the effect is observed in the absence of LPS binding protein, necessary for the action of low concentrations of LPS.

Bee venom sPLA_2_-III (structurally related to human group III) and human recombinant sPLA_2_-V were from Cayman (Tallinn, Estonia). Rapamycin, pyrazole pyrimidine-type 2 (PP2), porcine sPLA_2_-IB, LPS, both anti-rabbit and anti-mouse fluorescein isothiocyanate (FITC) secondary antibodies, FITC-dextran and other chemicals were from Sigma Chemical Co. (St. Louis, MO, USA.). PD98059 and AG1478 inhibitors were from Tocris Biosciece (Bristol, UK). Policlonal anti-heparin-binding epidermal growth factor (HB-EGF) neutralizing antibody and the inhibitors GM6001, chloromethylketone (CMK) and TNFα proteinase inhibitor-1 (TAPI-1) were from Calbiochem (San Diego, CA, USA). Rabbit anti-mitogen-activated protein kinase (MAPK) was from Zymed Laboratories (San Francisco, CA, USA). Rabbit antibody phosphorylated (phospho)-ERK1/2 (Thr202/Tyr204), phospho-S6 ribosomal protein (Ser235/236) and phospho-P70S6 kinase (Thr389) were from Cell Signaling Technology, Inc. (Danvers, MA, USA). The Rabbit phosphor-Src (Ser473), phospho-EGF (Tyr1173), phospho-EGF (Tyr845), anti-actin, and COX-2 antibodies were from Santa Cruz Biotechnology Inc. (Santa Cruz, CA, USA). Hybond-P membrane was from Amersham Biosciences (GE Healthcare Europe GmbH, Barcelona, Spain). DMEM and the cell culture supplements, including FCS, were purchased from Gibco BRL (Burlington, Canada).

### Cell culture

BV-2 murine microglia cells, a generous gift from Dr JR Bethea (University of Miami School of Medicine, Miami, Florida, USA), were cultured at 37°C in a humidified atmosphere of 5% CO_2_ in high sucrose DMEM, supplemented with 100U/ml penicillin, 100 μg/ml streptomycin, 50 μg/ml gentamicin, 2 mM glutamine, and 10% heat-inactivated fetal calf serum (FCS).

Primary microglia-enriched cultures were obtained from primary mixed glial cultures from 2- to 4-day-old neonatal C57BL/6 mice. To obtain mixed glial cultures, cerebral cortices were dissected, carefully stripped of their meninges, and digested with 0.25% trypsin-EDTA solution (Invitrogen, Life Technologies S.A., Madrid, Spain) for 25 minutes at 37°C. Trypsinization was stopped by adding an equal volume of culture medium, to which 0.02% deoxyribonuclease I (Sigma) was added. The culture medium consisted of DMEM-F-12 nutrient mixture supplemented with 10% FCS, 0.1% penicillin-streptomycin, and 0.5 μg/mL amphotericin B (Fungizone, Invitrogen). Cells were pelleted (5 minutes, 200 g), re-suspended in culture medium, and brought to a single cell suspension by repeated pipetting followed by passing through a 105 μm-pore mesh. Cells were seeded at a density of 3.5 × 10^5^ cells/ml (1.2 × 10^5^ cells/cm^2^) and cultured at 37°C in a 5% CO_2_ humidified atmosphere. Medium was replaced every 5 to 7 days. Microglial cultures were prepared by the mild trypsinization method previously described by Saura *et al*. [34]. Briefly, after 19 to 21 days *in vitro*, mixed glial cultures were treated for 30 minutes with 0.06% trypsin in the presence of 0.25 mM EDTA and 0.5 mM Ca^2+^. This resulted in the detachment of an intact layer of cells containing virtually all the astrocytes, leaving a population of firmly attached cells identified as > 98% microglia. The microglial cultures were treated 24 h after isolation by this procedure. Experiments were carried out in accordance with the Guidelines of the European Union Council (86/609/EU), following the Spanish regulations (BOE 67/8509-12, 1988) for the use of laboratory animals, and approved by the Animal Ethics Committee of the Universidad de Valladolid. Cultures were found to be 99% microglia by staining with FITC-conjugated Griffonia (Bandeiraea) simplicifolia lectin I-B4 isolectin (1:100, Sigma St. Louis, MO, USA), a lectin that recognizes microglia, and an antibody against glial fibrillary acidic protein (Santa Cruz Biotechnology, Inc., Santa Cruz, CA), to identify astrocytes.

Primary and immortalized microglial cells were serum-starved 24 h before the experiments, and then were stimulated for different times, as indicated, in the presence or absence of inhibitors.

### Proliferation assay

Cell proliferation was quantified using the Promega kit, Cell Titer 96^R^Aqueous One Solution Cell Proliferation Assay (Promega Corporation, Madison, Wisconsin, USA, according to the manufacturer's recommendations. Briefly, primary and immortalized BV-2 microglial cells were seeded in 96-well tissue culture plates and serum-starved for 24 h. Then, cells were treated in quadruplicate with the stimuli, in the presence or absence of the indicated inhibitors. After 24 h of incubation, formazan product formation was assayed by recording the absorbance at 490 nm in a 96-well plate reader. The results were expressed as optical density (OD) values, as an assessment of the number of metabolically active cells. Microglia cell viability was also assessed by trypan blue exclusion.

### Western blot analysis

After treatment, cells were washed twice with PBS and harvested in Laemmli SDS sample buffer. Protein extracts were separated by SDS-PAGE and transferred to polyvinylidene difluoride membranes, which were incubated for 18 h at 4°C with the indicated antibodies, including ERK 1/2, p-ERK1/2, p-P70S6K, p-rS6, COX-2 and actin. After washing with Tris-Tween buffered saline (TTBS), a 1:2.000 (v/v) dilution of horseradish peroxidase-labeled immunoglobulin (IgG) was added at room temperature for 30 h. The blots were developed using enhanced chemiluminescence.

### Flow cytometric analysis

BV-2 cells, 5 × 10^6^/flask, were treated with 1 μg/ml of sPLA_2_-IIA for different periods of time at 37°C. Cells to be analyzed for expression of epidermal growth factor receptor (EGFR) were fixed in a mixture of 4% paraformaldehyde and 0.2% Triton X-100 in PBS for 15 minutes at room temperature, before incubation with FITC-conjugated anti-mouse EGFR antibody for 1 h at 4°C, as previously described [35]. For EGFR phosphorylation analysis, cells were fixed in 4% paraformaldehyde for 15 minutes, washed with PBS, permeabilizaed with 0.3% Triton X-100 for 5 minutes, washed, incubated with anti-phospho EGFR (Tyr1173) or EGFR (Tyr845) antibody for 1 h at 4°C, and then with an FITC-labelled secondary antibody for 45 min at 4°C. After washing, the cells were analyzed with a Flow Cytometer (Gallios^TM^; Beckman Coulter, Brea, CA, USA). Data analysis was performed using WinMDI 2.7 software (Scripps Institute, La Jolla, CA, USA).

### Induction of apoptosis

Jurkat T cells were cultured in RPMI 1640 with 10% FBS at 37°C in 5% CO_2_. Apoptosis was induced in Jurkat T cells (10^6^ cells/ml) by overnight exposure to 400 μM H_2_O_2_ in serum-free RPMI medium. To distinguish between cells in the early or late stages of apoptosis, staining with Annexin V-FITC was combined with propidium iodide (PrI) staining. Afterwards, cells were immediately analyzed by flow cytometry (Gallios^TM^; Beckman Coulter, USA). Cells in the early stage of apoptosis were negative for PrI but stained with Annexin V-FITC, whereas in the late stage apoptotic cells stained for both PrI and Annexin V-FITC. Jurkat T cells treated in this way were about 90% late-stage apoptotic cells.

### Phagocytosis assays

#### Phagocytosis of particles

Microglial cells seeded in 96-well plates or in 25-mm^2^ flasks were incubated with medium, 1 μg/ml of sPLA_2_-IIA, 100 UI/ml of interferon-γ (IFNγ) at 37°C for 24 h, in the presence or absence of the indicated inhibitors. After 24 h, the phagocytic ability of the cells was measured using FITC-dextran as a tracer [36]. Briefly, cells were exposed to 0.1 mg/ml of FITC-labelled dextran (MW 40,000) for 2 h. Non-internalized particles were removed by vigorously washing three times with cold PBS (pH 7.4) prior to measuring fluorescence at 480 nm excitation and 520 nm emission on either a Flow Cytometer (Gallios^TM^; Beckman Coulter, USA) or a Fluoroskan multiwell plate reader (TECAN Genios Pro; Tecan Group Ltd, Männedorf, Switzerland). As a background, the cultures without FITC-dextran were used (blank wells). Each culture condition was performed in quadruplicate, and three independent experiments were performed. To visualize the internalized dextran, cells were also analyzed on a Leica TCS SP5X confocal microscope with a ×60 oil objective.

#### Phagocytosis of apoptotic cells (efferocytosis)

Phagocytic assays were performed on BV-2 cells (effector cells) after 24 h incubation in the presence of the inflammatory stimuli. Apoptotic Jurkat T cells were used as target cells. Briefly, PrI-labeled apoptotic Jurkat T cells were added to the BV-2 cells at a 8 to 10:1 ratio (apoptotic cell to BV-2) and incubated at 37°C in 5% CO_2_ for 2 h in DMEM medium. Then, BV-2 cells were washed gently with cold PBS and trypsinized by incubating them with a solution 0.25% trypsin/EDTA for 5 minutes to remove uningested cells. Afterwards, cells were fixed, stained with a PE-conjugated-CD68 antibody and analyzed by flow cytometry. PE fluorescence was analyzed in FL2 (555 to 600 nm), while red fluorescence from PrI was analyzed in FL3 (555 to 600 nm). To quantify phagocytosis, PrI fluorescence was analyzed only in the cell populations exhibiting PE-CD68 positive staining (BV-2 microglia cells). The BV-2 microglia cells were positive for PrI fluorescence only if they had ingested PrI-labeled Jurkat T cells. To confirm efferocytosis, a Leica TCS SP5X confocal microscope was used with the Leica LAS AF acquisition software and a ×60 oil objective. For confocal microscopy, BV-2 cells were plated onto 12-mm round cover slips and stained with an Alexa-fluor-CD11b antibody. We used 4',6'-diamidino-2-phenylindole hydrochloride (DAPI) to identify nuclei in BV-2 cells.

### Statistical analysis

All data were expressed as the mean ± SD and analyzed by one-way ANOVA followed by post-hoc comparisons (Bonferroni test) using the GraphPad Prism Version 4 software (San Diego, CA, USA). *P* < 0.05 was considered statistically significant.

## Results

### sPLA_2_-IIA triggers microglial proliferation

**Figure 1:**
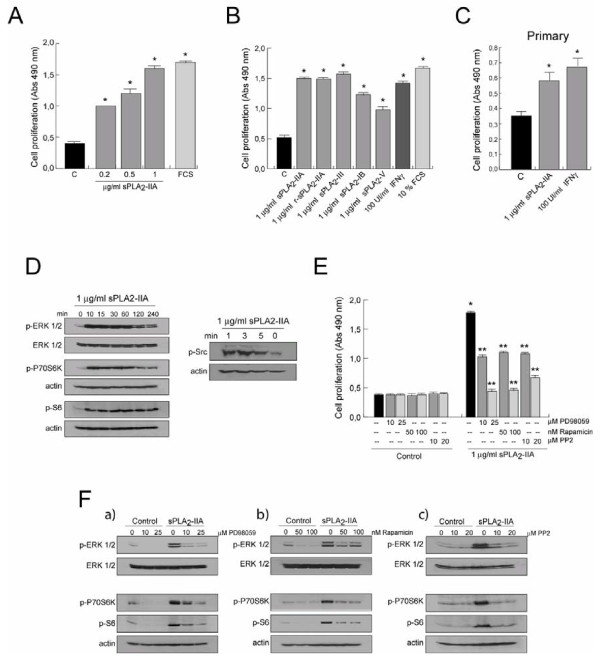
**sPLA**_**2**_**-IIA modulates proliferation in microglial cells.** BV-2 cells were stimulated with different doses of sPLA_2_-IIA and 10% of FCS **(A)**, or with the indicated stimuli **(B)**. Primary microglial cells were stimulated with 1 μg/ml of sPLA_2_-IIA or 100 UI/ml of IFNγ **(C)**. After 24 h of incubation at 37°C, cell proliferation was investigated and expressed as optical density (OD) values ± SD. Values are the average of three separate experiments in quadruplicate (*P < 0.001 vs. control cells). **(D)** BV-2 microglial cells were incubated with sPLA_2_-IIA for different times. Cell lysates were collected and subjected to western blot analysis using Abs against p-Src, ERK 1/2, p-ERK1/2, p-P70S6K, p-rS6 and actin. **(E)** BV-2 cells were treated at 37°C for 30 minutes in the presence or absence of the indicated inhibitors, and then were stimulated with 1 μg/ml of sPLA_2_-IIA. After 24 h of incubation, cell proliferation was investigated and expressed as OD values ± SD. Values are the average of three separate experiments in quadruplicate (*P < 0.001 vs. control cells, **P < 0.001 vs. sPLA_2_-IIA-treated cells). **(F)** BV-2 cells were treated as in (D). After 15 minutes stimulation, whole cell lysates were extracted and protein phosphorylation was assessed by western blotting using p-ERK, p-P70S6 and p-rS6 antibodies. Membranes were always stained with Ponceau S as a loading control.

A great deal of attention has recently focused on the cytokine-like actions of sPLA_2_-IIA and its input to inflammation-associated diseases. Having been found highly expressed in several CNS pathological conditions, we hypothesized that sPLA_2_-IIA might act as a cytokine-like modulator on brain-resident immune cells. To test this possibility, we examined whether sPLA_2_-IIA could induce some of the hallmarks of activated microglia. We used the immortalized mouse microglial cell line BV-2 as an *in vitro* model to mimic the microglial activation observed in neurodegenerative disorders — such cells have been proven to reproduce the behavior of primary microglia and do not express endogenous sPLA_2_-IIA [14,37-39]. Serum-starved BV-2 cells were stimulated for 24 h with the indicated concentrations of sPLA_2_-IIA, and its effect on the proliferative activity of the cells was evaluated with a colorimetric assay. Our results revealed that sPLA_2_-IIA markedly stimulated cell proliferation in a dose-dependent manner and reached a 3-fold increase (*P* < 0.001) when stimulated with 0.5 μg/ml of sPLA_2_-IIA, as compared with unstimulated cells (Figure 1A). The dose inducing the maximal change, 1 μg/ml, was used for all subsequent experiments. We also found a strong mitogenic response to other secreted PLA_2_s (1 μg/ml), as well as to the well known inducer/amplifier of microglia pro-inflammatory functions, IFNγ (100 UI/ml) (Figure 1B). Furthermore, as shown in Figure 1C, primary microglial cultures also responded to the addition of sPLA_2_-IIA and IFNγ with a modest but significant (*P* < 0.001) increase in cell proliferation (Figure 1C). 

This effect on growth was paralleled by the activation/phosphorylation of key proteins involved in cell survival and proliferation such as ERK, P70S6K and rS6. Activated forms of these proteins from whole cell lysates were monitored using specific anti-phospho antibodies that recognize only their activated/phosphorylated form. To determine whether the mTORC1 pathway was activated following sPLA_2_-IIA stimulation, we used an antibody that detects phosphorylation of P70S6K on threonine 389, a site well known to be selectively phosphorylated by mTORC1 and widely used to monitor mTORC1 activation.

As shown in Figure 1D, sPLA_2_-IIA treatment induced a rapid and sustained increase in ERK, P70S6K and rS6 phosphorylation in BV-2 cells. This effect was blocked in the presence of specific pharmacological inhibitors, including PD98059 (MEK inhibitor), rapamicin (mTOR inhibitor) and PP2 (Src kinase inhibitor), which also affected the proliferative response (Figure 1E, F). Thus, ERK and mTORC1 are key components of the intracellular signals regulating cell growth.

### Involvement of epidermal growth factor receptor (EGFR) transactivation in sPLA_2_-IIA-enhanced microglial cell proliferation

**Figure 2:**
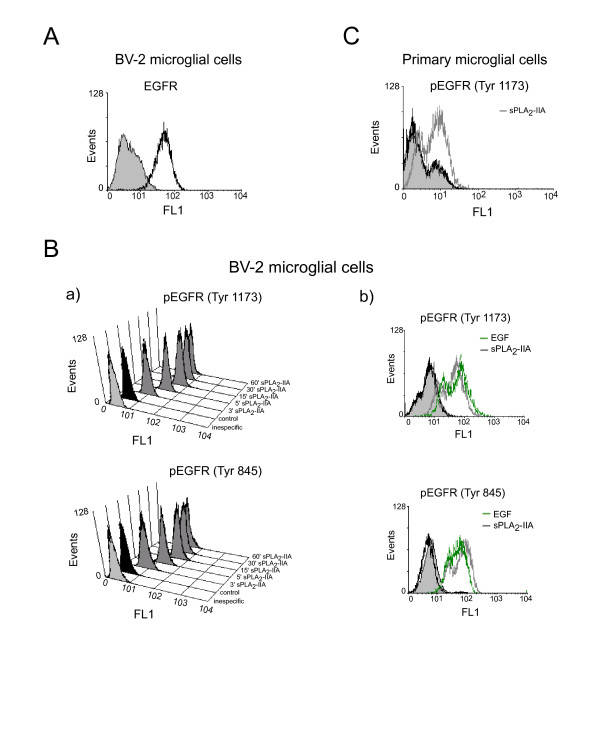
**sPLA**_**2**_**-IIA induces epidermal growth factor receptor (EGFR) transactivation in microglial cells.(A)** EGFR expression on untreated BV-2 cells was analyzed by flow cytometry (open black curves) and compared with isotype controls (solid grey curves). **(B)** Flow cytometry analysis of phospho-EGFR levels: **a)** BV-2 cells were untreated (solid black curves) or treated with 1 μg/ml of sPLA_2_-IIA (solid dark grey curves) for different times; **b)** BV-2 cells were untreated (open black curves), treated with 1 μg/ml of sPLA_2_-IIA (open dark grey curves), or treated with 0.4 μM of EGF (open green curves) for 15 minutes, and compared with isotype controls (solid light grey curves). **(C)** Flow cytometry analysis of phospho-EGFR levels in primary microglial cells. Cells untreated (open black curves) and treated with 1 μg/ml of sPLA_2_-IIA for 15 (open dark grey curves). Solid light grey curves show negative controls in which the primary antibody was omitted. Results shown are representative of three independent experiments.

Next, we analyzed whether sPLA_2_-IIA-induced cell proliferation involves EGFR signaling, since transactivation of this receptor is a crucial signaling mechanism for controlling cell survival, migration and proliferation. Functional expression of EGFR in microglial cells has been previously described [40], and a flow cytometry analysis revealed that resting BV-2 cells also constitutively express it (Figure 2A). After that, we investigated whether sPLA_2_-IIA treatment caused tyrosine phosphorylation of EGFR at Tyr-845, as well as at Tyr-1173 (a major autophosphorylation site), by using anti-phospho-specific antibodies and flow cytometry analysis. As shown in Figure 2B.a, a rapid and sustained phosphorylation of EGFR at both Tyr-1173 and Tyr-845 was detected in BV-2 cells upon phospholipase stimulation. Phosphorylation of Tyr-845 is believed to stabilize the receptor activation loop and is required for the mitogenic function of the receptor, whereas phosphorylation of Tyr-1173 is involved in MAPK activation. In addition, EGFR phosphorylation in response to sPLA_2_-IIA was similar in extent to that observed in response to EGF (used as a positive control). Studies on primary microglial cells also showed EGFR phospharylation at Tyr-1173 upon sPLA_2_-IIA treatment (Figure 2C). These results indicate that sPLA_2_-IIA is able to cause transactivation of EGFR in microglial cells. 

**Figure 3:**
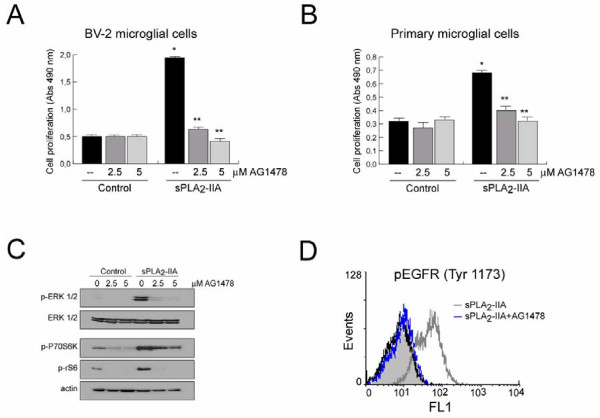
**sPLA**_**2**_**-IIA-induced proliferation in microglial cells is dependent on epidermal growth factor receptor (EGFR) transactivation.(A,B)** Primary and immortalized BV-2 microglial cells were stimulated with 1 μg/ml of sPLA_2_-IIA in the absence or presence of AG1478 at 37°C. After 24 h, cell proliferation was investigated and expressed as optical density values ± SD. Values are the average of three separate experiments in quadruplicate (*P < 0.001 vs. control cells, **P < 0.001 vs. sPLA_2_-IIA-treated cells). **(C)** After 15 minutes, ERK 1/2, P70S6 and rS6 phosphorylation were identified in the cell lysates of BV-2 microglial cells by western blot. Membranes were stained with Ponceau S as a loading control. **(D)** BV-2 cells were stimulated with 1 μg/ml of sPLA_2_-IIA in the absence or presence of 2.5 μM of AG1478 for 15 minutes. Flow cytometry analysis of phospho-EGFR levels in untreated (open black lines), sPLA_2_-IIA-treated (open dark grey lines) and AG1478 + sPLA_2_-IIA-treated (open blue lines) cells. Solid light grey curves show negative controls in which the primary antibody was omitted. Results shown are representative of three independent experiments.

Next, to determine whether EGFR transactivation is required for sPLA_2_-IIA-induced mitogenic signals, we pre-incubated primary and immortalized BV-2 cells in the presence of different doses of the selective EGFR tyrosine kinase inhibitor, AG1478. We found that the presence of the inhibitor diminished the proliferative response induced by 24 h of phospholipase stimulation (Figure 3A, B) in a dose-dependent manner. The activation and phosphorylation of the key signaling proteins ERK, P70S6K and rS6 (Figure 3C), as well as EGFR phospholylation at Tyr-1173 (Figure 3D) was fully abolished in AG1478-pretreated BV-2 cells. The presence of AG1478 only partially suppressed phosphorylation of Tyr-845 (data not shown). These findings demonstrate that EGFR transactivation accounted for sPLA_2_-IIA-promoted cell proliferation and intracellular signaling in microglial cells, and suggest that EGFR phosphorylation initiated by sPLA_2_-IIA requires its intrinsic kinase activity.

**Figure 4:**
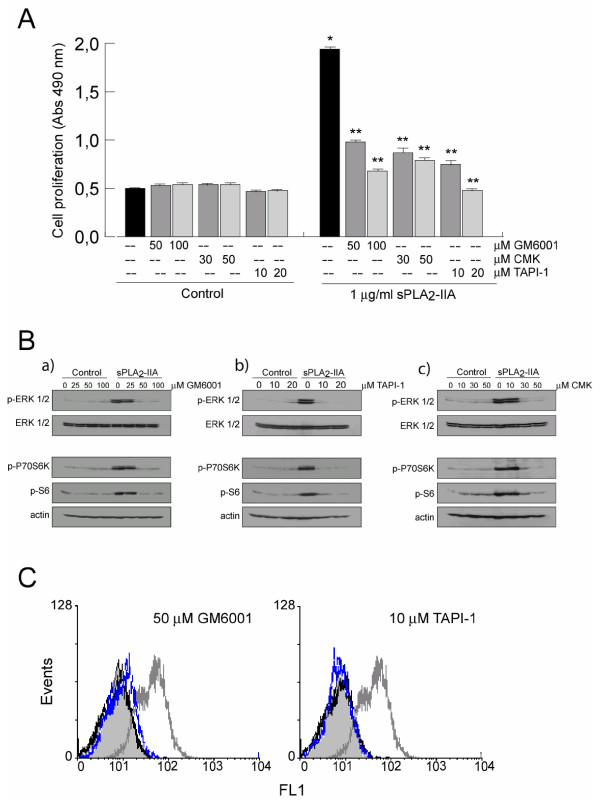
**Matrix metalloproteinases inhibitors modulate the proliferative response induced by sPLA**_**2**_**-IIA in BV-2 cells.** BV-2 cells were treated for 30 minutes at 37°C in the presence or absence of the indicated inhibitors, and then were stimulated with 1 μg/ml of sPLA_2_-IIA. **(A)** After 24 h, cell proliferation was investigated and expressed as optical density. values ± SD. Values are the average of three separate experiments in quadruplicate (*P < 0.001 compared to control cells, **P < 0.001 compared to sPLA_2_-IIA-treated cells). **(B)** After 15 minutes, ERK 1/2, P70S6 and rS6 phosphorylation were identified in the cell lysates by western blot. Membranes were stained with Ponceau S as a loading control. **(C)** Phosphorylation levels of EGFR on untreated (open black curves), 1 μg/ml of sPLA_2_-IIA-treated (open dark grey curves), and MMP inhibitors + 1 μg/ml of sPLA_2_-IIA-treated (open blue curves) BV-2 cells were analyzed by flow cytometry and compared with isotype controls (solid grey curves). Results shown are representative of three independent experiments.

Several lines of evidence have suggested that transactivation of EGFR may be mediated via metalloproteinases (MMP) by extracellular release of EGFR ligands, such as transforming growth factor (TGF), amphiregulin and heparin-binding EGF-like growth factor (HB-EGF), from the cell membrane. To identify the potential underlying mechanisms linking sPLA_2_-IIA-induced proliferation and EGFR transactivation, microglia cells were then pre-incubated for 30 minutes with either the general matrix metalloproteinase inhibitor GM6001 (commonly used to inhibit ectodomain shedding), the disintegrin and metalloproteinase domain (ADAM) inhibitor, TAPI-1 (TNF-α proteinase inhibitor-1) or the furin inhibitor CMK (utilized to block ADAM zymogens activation), and then challenged with 1 μg/ml of sPLA_2_-IIA for 24 h. As shown in Figure 4A, the mitogenic ability of the sPLA_2_-IIA was significant reduced, or even abolished, in the presence of the mentioned inhibitors. Subsequently, we examined the effect of these inhibitors on the phosphorylation of ERK, P70S6K and rS6 proteins. As shown in Figure 4B.a,b,c, pre-treatment of cells with these inhibitors completely blocked the sPLA_2_-IIA effect on the phosphorylation of the studied proteins. In addition, by flow cytometry analysis, we also found that the presence of GM6001 and TAPI-1 successfully reduced the EGFR phosphorylation triggered by sPLA_2_-IIA (Figure 4C). Interestingly, pre-treatment with the selective inhibitors PD98059 (25 μM) and rapamycin (50 nM), did not affect EGFR phosphorylation induced by sPLA_2_-IIA, whereas it was fully prevented by the presence of Src kinase inhibitor, PP2 (10 μM) (data not shown), suggesting that EGFR phosphorylation can occur by multiple mechanisms. We also used the highly selective inhibitor of MEK1/2, U0126, and we found that while ERK phosphorylation induced by sPLA_2_-IIA was completely abolished by the presence of 5 and 10 μM of U0126, phosphorylation of EGFR both at Tyr1173 and at 845 was not affected (data not shown). These results also imply that ERK and mTOR pathways are downstream targets of EGFR signaling.

### sPLA_2_-IIA induces a proliferative response in microglial cells via an epidermal growth factor receptor (EGFR)-ligand-dependent mechanism

**Figure 5:**
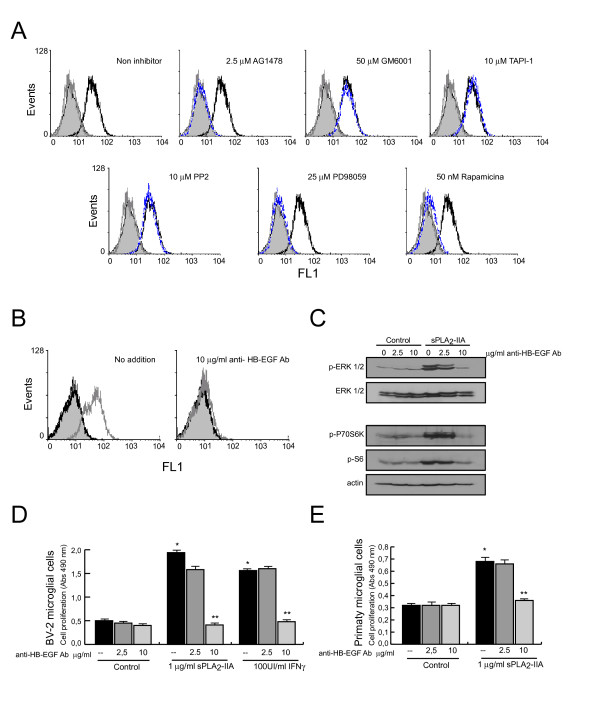
**Involvement of heparin-binding epidermal growth-like growth factor (HB-EGF) shedding in sPLA**_**2**_**-IIA-induced a proliferative response.** BV-2 cells were treated with the indicated inhibitors for 30 minutes at 37°C and then incubated with 1 μg/ml of sPLA_2_-IIA for 5 minutes. **(A)** HB-EGF expression was analyzed by flow cytometry: untreated cells (open black curves) were compared with sPLA_2_-IIA-treated cells in the absence (open dark grey curves) or presence of the indicated inhibitors (open blue curves). Solid light grey curves show negative controls in which the primary antibody was omitted. (B, C) BV-2 cells were incubated for 30 minutes at 37°C in the presence of the indicated dose of an anti-HB-EGF neutralizing antibody, and then stimulated with 1 μg/ml of sPLA_2_-IIA. **(B)** After 15 minutes incubation, phosphorylation of EGFR was analyzed by flow cytometry: untreated cells are represented by open black curves, whereas sPLA_2_-IIA-treated cells both in the absence and in the presence of the neutralizing Ab are represented by open dark grey curves. Solid grey curves represent isotype controls. **(C)** After 15 minutes, whole cell lysates were extracted and protein phosphorylation was assessed by western blotting using p-ERK, p-P70S6, p-rS6 and actin antibodies. Membranes also were stained with Ponceau S as a loading control. Results shown are representative of three independent experiments. **(D)** Primary and immortalized BV-2 microglial cells were stimulated as indicated in the presence or absence of an anti-HB-EGF neutralizing antibody. After 24 h, cell proliferation was investigated and expressed as optical density values ± SD. Values are the average of three separate experiments in quadruplicate (*P < 0.001 compared to control cells, **P < 0.001 compared to sPLA_2_-IIA-stimulated cells).

Among the various EGFR ligands that could be processed by proteolysis, we focused on HB-EGF, because it is both a leading molecule linked to ligand shedding and EGFR transactivation, and pro-HB-EGF is a target of ADAMs enzymes. To determine whether HB-EGF contributes to sPLA_2_-IIA-induced cell growth and signaling in BV-2 cells, we first examined its cell surface expression by flow cytometry analysis using an ectodomain-specific antibody. As shown in Figure 5A, BV-2 microglial cells constitutively express pro-HB-EGF and their stimulation with 1 μg/ml of sPLA_2_-IIA results in a rapid 5-minute reduction of its levels in the cell surface. This reduction in cell surface content of endogenous pro-HB-EGF, while completely unaffected by the presence of AG1478 (2.5 μM), was fully prevented by pre-treating the cells with the non-selective metalloproteinase inhibitor GM6001 (50 μM) or the ADAMs inhibitor TAPI-1 (10 μM), pointing to an ADAMs-mediated mechanism by which sPLA_2_-IIA-treatment might cause the shedding of pro-HB-EGF on BV-2 cells. In addition, inhibition of the ERK and mTOR pathways with PD98059 or rapamicyn, respectively, did not alter the pro-HB-EGF cell surface expression levels of sPLA_2_-IIA-stimulated cells. In contrast, the presence of the Src kinase inhibitior PP2 completely blocked sPLA_2_-IIA-induced HB-EGF release. Next, we examined the contribution of HB-EGF shedding to sPLA_2_-IIA-indued EGFR transactivation and signaling by pre-incubating the cells for 30 minutes with a polyclonal anti-HB-EGF neutralizing antibody, which prevents binding of HB-EGF to the extracellular domain of the EGFR. As shown in Figure 5B and C, the presence of the neutralizing antibody completely prevented sPLA_2_-IIA-induced tyrosine phosphorylation of EGFR, ERK, P70S6K and rS6. Moreover, we found that the presence of the neutralizing antibody abrogated the ability of the phospholipase to enhance primary and immortalized BV-2 cell proliferation (Figure 5D and E). Interestingly, IFNγ induced a mitogenic response in BV-2 cells that was also HB-EGF-dependent. These data support the hypothesis that the EGFR pro-ligand HB-EGF is required for sPLA_2_-IIA to stimulate cell growth, and for activation of key intracellular signaling pathways.

### sPLA_2_-IIA treatment enhances phagocytosis and efferocytosis in BV-2 microglia cells

**Figure 6:**
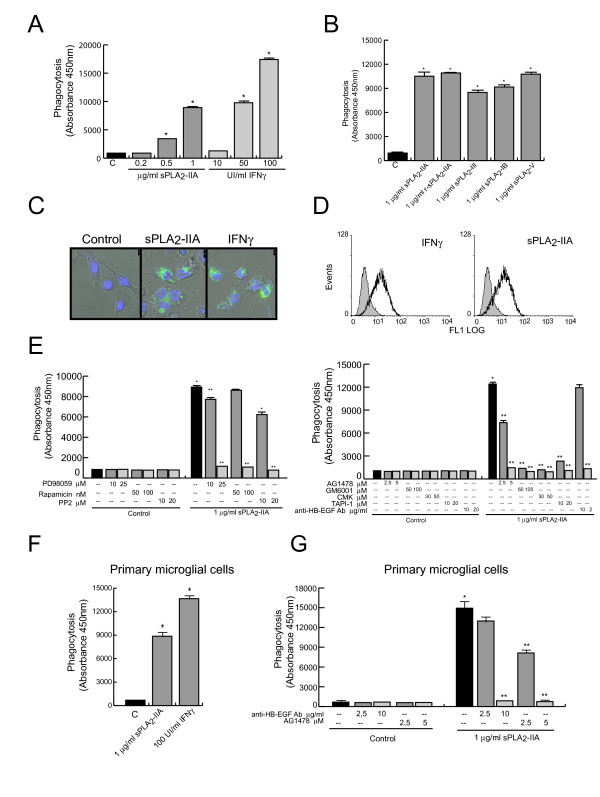
**sPLA**_**2**_**-IIA enhances phagocytosis on microglial cells.** sPLA_2_-IIA induces fluorescein-isothiocyanate (FITC)-dextan beads uptake in microglial cells. BV-2 microglial cells were treated with different doses of sPLA_2_-IIA and IFNγ **(A)**, or with 1 μg/ml of different sPLA_2_ isoforms **(B)** for 24 h at 37°C. Primary microglial cells were treated with 1 μg/ml of sPLA_2_-IIA and IFNγ **(F)**. Then, cells were co-incubated with FITC-dextan beads and quantified using a fluorescent plate reader, as detailed in Methods. Results are the average of three separate experiments in quadruplicate and are expressed as the mean of the fluorescent intensity values ± SD (*P < 0.001 vs. control cells). **(C)** Images with a Leica TCS SP5X confocal microscope, under a ×60 oil objective, confirm that fluorescent dextran associated with BV-2 microglia is internalized by the cells and not only bound to the cell surface. **(D)** BV-2 cells were cultured in the absence or presence of 1 μg/ml of sPLA_2_-IIA or 100 UI/ml of IFNγ for 24 h at 37°C. Then, they were incubated with FITC-dextran for 1 h and analyzed by flow cytometry. Histograms represent one experiment out of three. Solid grey curves represent unspecific endocytosis and open black curves endocytosis of cells cultured as indicated. **(E,G)** Primary and immortalized BV-2 microglial cells were pretreated with the indicated inhibitors for 30 minutes. Cells were then treated with 1 μg/ml of sPLA_2_-IIA at 37°C for 24 h followed by co-incubation with FITC-dextran for 30 minutes at 37°C. Phagocytosis was quantified using a fluorescent plate reader as described under Methods. Values ± SD are the average of three separate experiments in quadruplicate (*P < 0.001 vs. control cells, **P < 0.001 compared to sPLA_2_-IIA stimulated cells).

To determine whether sPLA_2_-IIA-induced changes in growth are extended to other functional aspects of microglia, we studied the effect of sPLA_2_-IIA on the phagocytic capacity of BV-2 cells. Microglial cells were exposed to sPLA_2_-IIA for 24 h, and phagocytosis assays were carried out by incubating activated microglial cells with either FITC-labeled dextran beads (as measures of non-specific phagoytosis) or apoptotic Jurkat cells (as measures of disease-relevant processes of phagocytosis). To quantify phagocytosis of fluorescent particles/cells a flow cytometer and a microplate fluorescence reader were used. IFNγ-treated BV-2 cells were taken as the positive control in the above experiment. As shown in Figure 6A and F, cell stimulation with both sPLA_2_-IIA and IFNγ enhanced phagocytic function in both primary and immortalized BV-2 microglial cells. In a parallel set of experiments, the effect of sPLA_2_-IIA at the optimal dose of 1 μg/ml was compared with that of other secreted phospholipase A_2_ isoforms: sPLA_2_-III, -IB or -V, to clarify whether the action of sPLA_2_-IIA on microglial phagocytosis is a general property of the sPLA_2_ family. As shown in Figure 6B, we found that all tested phospholipases had a similar stimulatory effect on promoting microglial phagocytosis of dextran beads. To further confirm their internalization, confocal microscopy was used (Figure 6C). Representative confocal fluorescence images clearly demonstrated that the fluorescent dextran beads were taken up into the cytoplasm of BV-2 microglial cells. We also evaluated the uptake of FITC-labeled dextran beads using flow cytometry analysis (Figure 6D). Both sPLA_2_-IIA- and IFNγ-treated BV-2 cells showed higher intracellular levels of the labeled dextran beads in comparison to untreated cells. Interestingly, the presence of inhibitors targeting specific upstream and downstream signaling mediators of EGFR transactivation efficiently suppressed the phagocytic response induced by sPLA_2_-IIA (Figure 6E). Similar results were obtained in mouse primary microglia cells (Figure 6G).

**Figure 7:**
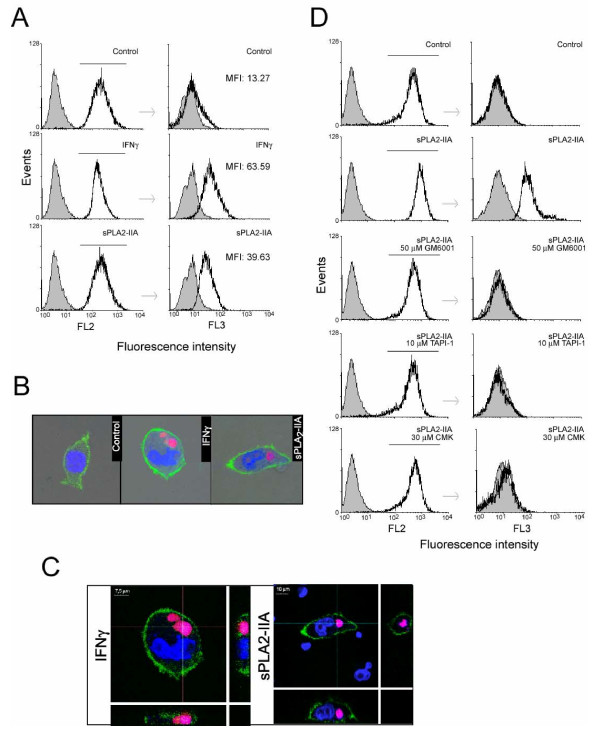
**sPLA**_**2**_**-IIA induces efferocytosis in BV-2 cells.** BV-2 cells were treated with 1 μg/ml of sPLA_2_-IIA or 100 UI/ml IFNγ for 24 h at 37°C, exposed to PrI-labelled apoptotic Jurkat cells for 2 h, and then stained with an anti-PE-CD68 antibody, as described under Methods. **(A)** Phagocytosis was analyzed by flow cytometry. FL2 fluorescence (left) shows BV-2 cells expressing CD68. The bar marks the population of BV-2 cells which was gated for detection of PrI-apoptotic cell uptake. FL3 fluorescence (right) shows the red fluorescence (PrI-apoptotic cells) in PE-CD68-labelled cells (on the gated BV-2 cells). As a measure of the phagocytosed cells, MIF values are shown. **(B)** Phagocytosis was also studied by confocal microscopy (final magnification ×60). Co-cultures of PrI-apoptotic cells with either resting or stimulated BV-2 cells were fixed and stained with Alexa-fluor-CD11b and DAPI. Laser confocal images confirm that fluorescent apoptotic cells associated with BV-2 microglia are internalized by the cells and not only bound to the cell surface. **(C)** Confocal images with reconstructed orthogonal projections presented as viewed in the x-z plane (bottom) and y-z plane (right): Fluorescent apoptotic cells (stained with PrI) are shown to be internalized by BV-2 microglia. (DAPI-labelled nuclei, blue). Scale bars: 10 μm in sPLA2-treated cells, and 7.5 μm in IFNγ-treated cells. **(D)** BV-2 cells were treated with the indicated inhibitor for 30 minutes before sPLA_2_-IIA stimulation and mixing with the apoptotic cells. Phagocytosis was analyzed as in (A). In all the histograms, the solid grey curve represent the resting/control cells. The results are representative of three independent experiments.

Next, we investigated the potential for BV-2 cells to engulf apoptotic cells (efferocytosis) and the effect of sPLA_2_-IIA in this system (Figure 7). As described in Methods, apoptotic Jurkat T cells were loaded with PrI to visualize engulfed T cells within microglial cells, and BV-2 cells were immunostained with CD68-PE. Jurkat T cells were treated for 18 h with 400 μM of H_2_O_2_ and apoptosis was confirmed by an annexin-V assay (data not shown). Apoptotic Jurkat T cells were then added to a culture of BV-2 cells-treated under different conditions with a ratio of Jurkat to BV-2 cells of 8:1. After 2 h incubation, the co-culture was analyzed by flow cytometry to quantify cell uptake. As shown in Figure 7A, we observed very little phagocytosis under control conditions where BV-2 cells were resting. However Jurkat engulfment increased significantly when BV-2 cells were pre-treated for 24 h with 1 μg/ml of sPLA_2_-IIA or 100 UI/ml of IFNγ, as increasing number of microglia cells showed FL3-fluorescence-positive signals. In a separate experiment, the cells were also stained with DAPI and studied using a confocal microscope to visually confirm the ingestion of apoptotic cells (Figure 7B). The orthogonal reconstruction images showed the spatial relation of ingested cells to the BV-2 cell nucleus (Figure 7C) and confirm that Jurkat cells were not merely bound to the cell surface.

In subsequent experiments, we examined whether transactivation of EGFR is also a key step for controlling sPLA_2_-IIA-mediated efferocytosis. Consistent with the signaling mechanism recruited by the secreted phospholipase to promote proliferation of BV-2, we found that the presence of the selective inhibitors GM6001, CMK and TAPI-1 also abolished the phagocytic response triggered by the sPLA_2_-IIA on microglial cells (Figure 6D), as it previously did on sPLA_2_-IIA-enhanced cell growth.

### sPLA_2_-IIA promotes synthesis and secretion of inflammatory mediators in BV-2 cells

**Figure 8:**
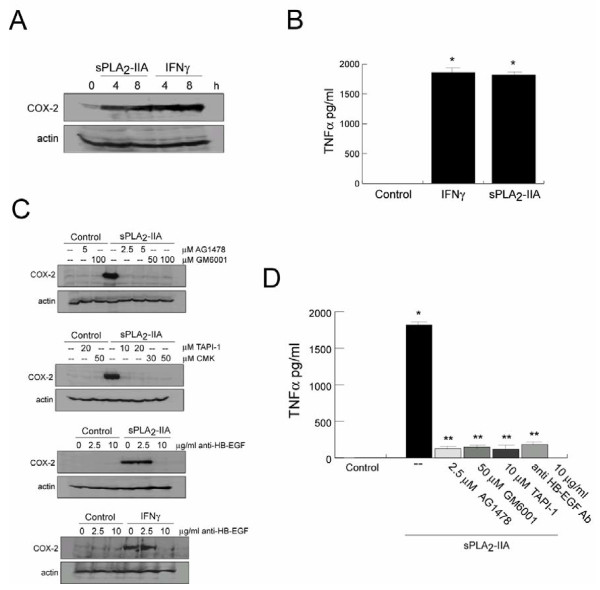
**sPLA**_**2**_**-IIA exhibits pro-inflammatory actions by stimulating TNFα and cycloxygenase-2 (COX-2) protein expression.** BV-2 microglial cells were incubated with 1 μg/ml of sPLA_2_-IIA or 100 UI/ml IFNγ at 37°C. **(A)** After 4 and 8 h of stimulation, COX-2 expression was identified in the cell lysated by western blot. Membranes were also stained with Ponceau S as a loading control. **(B)** After 24 h, the presence of TNFα in the cell culture medium was quantified by commercial ELISA. In some experiments, COX-2 expression **(C)** and TNFα secretion **(D)** was evaluated from cells pretreated with the indicated inhibitors. Results shown are representative of three independent experiments. (*P < 0.001 vs. control cells, **P < 0.001 vs. sPLA_2_-IIA-treated cells).

Finally, we examined whether sPLA_2_-IIA could affect the expression levels of pro-inflammatory mediators in BV-2 microglia cells. Then, BV-2 cells were treated with the optimal concentration of 1 μg/ml of sPLA_2_-IIA or 100 UI/ml of IFNγ for 4 and 8 h, and the expression of COX-2 was examined in the cell lysate by western blot. Our results revealed that both treatments markedly induced the expression of the pro-inflammatory protein COX-2 (Figure 8A). We also measured the production of the cytokine TNFα using a commercial ELISA assay. We observed that in the supernatant of cells treated with sPLA_2_-IIA or IFNγ for 24 h, the levels of TNFα were significantly enhanced, compared with untreated cells which did not produce TNFα spontaneously (Figure 8B). In contrast, the release or accumulation of anti-inflammatory mediators, such as IL-10 was not detected in any of our culture conditions (data not shown).

Lastly, we further examined whether blockage of EGFR signaling at different levels, as demonstrated in previous sections, affects the expression of these inflammatory proteins induced by sPLA_2_-IIA. Figure 8C and D show that sPLA_2_-IIA-induced up-regulation of COX-2 and secretion of TNFα was significantly inhibited by the presence of the inhibitors AG1478, GM6001, TAPI-1 and CMK, as well as by the polyclonal anti-HB-EGF antibody. Similarly, IFNγ-induced COX-2 expression was also abrogated by the presence of the neutralizing anti-HB-EGF antibody.

All these studies clearly pointed to a crucial role of EGFR transactivation, through MMP-mediated cleavage of mature forms of EGFR ligands, in the signaling and functional activity of the sPLA_2_-IIA.

## Discussion

**Figure 9:**
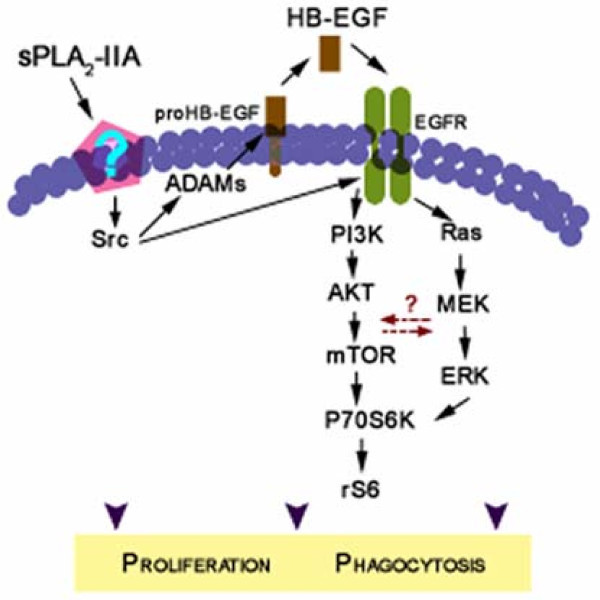
**Schematic representation of the signaling pathways involved in sPLA**_**2**_**-IIA-induced activation of microglial cells.** sPLA_2_-IIA, via Src-dependent activation, stimulates the shedding of the epidermal growth factor receptor (EGFR) ligand, heparin-binding-EGF, which in turn activates the EGFR. Phosphorylated EGFR results in activation of ERK and Akt/mTOR pathways, and the whole cascade stimulates microglia proliferation and augments their phagocytic activity.

Microglia, the major cellular source and target of inflammatory mediators in the CNS, are key players in neuroinflammatory disorders. These cells contribute to both pathogenic neurodegeneration and beneficial neuroprotection depending on how microglia interprets the threat [3,4,41]. Therefore, it is crucial to identify the various endogenous and exogenous factors that serve to activate microglia, as well as the functional responses elicited by them. In the present study we confirmed that exogenous sPLA_2_-IIA induces microglial activation, evidenced by increased cell proliferation, stimulation of their phagocytic capabilities and robust production of inflammatory mediators such as COX-2 and TNFα. We used primary and immortalized murine microglial cells with a defective *Pla2 g2a* gene, which makes them unable to produce sPLA_2_-IIA [14], to exclude potential actions of the endogenous phospholipase, since sPLA_2_-IIA may modulate different cell functions depending on its cellular location [42]. In addition, we demonstrated that sPLA_2_-IIA regulates functions of activated microglia through EGFR transactivation by induction of pro-HB-EGF processing via an ADAMs-dependent mechanism. Moreover, ERK and mTOR are key components of the intracellular signaling switch that transduce EGFR activation into the aforementioned characteristic of the activated microglia phenotype (Figure 9). 

The importance of sPLA_2_-IIA in neurodegenerative diseases, especially in those associated with inflammatory processes has started to emerge in recent years. Several studies have shown an increase in the expression of sPLA_2_-IIA in reactive astrocytes both in experimental models of cerebral ischemia and in specific regions of human brains in AD associated with amyloid plaques [11,12,18,23,26]. It has been suggested that the interaction of astrocytes with Aβ and other inflammatory stimuli, such as IL-1β or TNFα, are responsible for this sPLA_2_-IIA induction which could be associated in the early inflammatory events. Although the ability of sPLA_2_-IIA to affect the functional activities and the survival or death of astrocytes, neurons and oligodendrocytes has been explored, this is the first study in which the effect of sPLA_2_-IIA on microglial cells has been addressed. Our interest in microglia owes to the fact that these cells, in conjunction with astrocytes, are responsible for coordinating inflammatory responses in the brain and elicit immune responses against pathological stimuli.

Several pro-inflammatory and immunoregulatory responses associated with certain secreted PLA_2_ types have been reported in previous studies. Thus, sPLA_2_-IIA induces differentiation of monocytes into monocyte-derived dendritic cells or alternatively activated macrophages [36]; both human and bee venom type III trigger maturity of dendritic cells, which is accompanied by up-regulation of surface markers and by an increase in their migratory and immunostimulatory capacity [43,44]. Furthermore, type V regulates phagocytosis on macrophages by modulating phagosome maturation [45]. sPLA_2_-IIA also enhances the expression of COX-2 in mast cells and promotes degranulation and cytokine release in human eosinophils, as well as up-regulation of certain surface activation markers [46]. In addition, sPLA_2_-IIA, IB, X and III elicit proliferative signals, *in vitro*, in several cell types [30,33,47-49], and type IIA has proven to be protective even against oxysterol-induced apoptosis in oligodendrocytes [50].

In this study we showed that sPLA_2_-IIA, as well as type III, IB and V, enhance the proliferative and phagocytic capacity of BV-2 microglia cells to a similar extent as IFNγ, one of the cytokines up-regulated in the brain in different disorders and a well-known inducer of an activated state in microglial cells. Focusing on type IIA actions, two kind of phagocytosis have been evaluated: phagocytosis of inert particles (as a measure of non-specific phagocytosis) and of apoptotic cells (as a measure of disease-relevant processes of phagocytosis). The ability of microglia to phagocytose inert material and apoptotic cells is critical for the clearance of pathogen/cell debris and dead cells under pathological conditions. We demonstrated that sPLA_2_-IIA increases the uptake of apoptotic Jurkat T cells as well as dextran beads, thus indicating that sPLA_2_-IIA from the microenvironment might contribute to the innate immune response on the CNS by modulating the phagocytic efficiency of microglial cells. These findings are in concordance with the responses reported for other CNS soluble factors, including IFNγ, as well as for various secreted sPLA_2_s on other myeloid-lineage cells [36,43,44].

To our knowledge, there are no studies, either *in vivo* or *in vitro,* describing production and secretion of sPLA_2_-IIA by microglial cells, while astrocytes have been identified as a key cellular source of sPLA_2_-IIA in the CNS under different pathological conditions [11,12,18,23,51]. Therefore, we propose that the sPLA_2_-IIA, once released by astrocytes, might act on the microglia, in a paracrine manner, to promote microglial activation and to further stimulate phagocytosis and production of inflammatory mediators such TNFα or COX-2 , thereby affecting the inflammatory environment of the brain and contributing to additional neuronal cell damage.

These results have led us to question the possible mechanisms - signaling molecules and receptors - underlying the functional effects of sPLA_2_-IIA. It has previously been reported that the biological activities induced by sPLA_2_s can be dependent on both enzymatic and nonenzymatic mechanisms. Whereas the ability of types X and III to stimulate cell growth has been found to be mostly dependent on their intrinsic catalytic activity, the mitogenic response induced by type IB and IIA seems to be unrelated to its enzymatic activity. Both an integrin-dependent [30] and an EGFR-dependent pathway [29] have been characterized as new sPLA_2_-IIA putative signaling mechanisms. In this study, we found that sPLA_2_-IIA induced a phenotype of activated microglia in BV-2 cells which is linked to the activation of the classical MAPK/ERK and mTOR/P70S6K pathways through MMP-dependent ectodomain shedding of the transmembrane precursor pro-HB-EGF and subsequent transactivation of the EGFR.

The EGFR is expressed ubiquitously in the mammalian brain, being detected in neurons and glia cells [40,52]. It has been hypothesized that EGFR activation is a master signal transduction pathway of the cellular activation process in response to different brain injuries and causes the characteristics of the reactive astrocyte/microglia phenotype [53-55]. Thus, activation of the EGFR pathway is responsible for the hypertrophy, proliferation and migration of reactive astrocytes, and perhaps of activated microglia, at the site of neural injury [40,56,57]. We have herein showed that sPLA_2_-IIA induces a sustained EGFR phosphorylation at Tyr 1176 and Tyr 845 residues that is abolished or diminished in the presence of the selective EGFR inhibitor, AG1478. To understand the mechanisms by which phospholipase causes EGFR phosphorylation, we used a general matrix metalloprotease inhibitor (GM6001) and an ADAMs inhibitor (TAPI-1), which are known to block the proteolytic cleavage of various membrane-anchored EGFR pro-ligands such as pro-EGF, pro-TGFα, pro-HB-EGF, and pro-amphiregulin. We have found that the presence of these inhibitors blocked the effect of sPLA_2_-IIA on EGFR phosphorylation as well as on ectodomain shedding of HB-EGF, suggesting a possible role of ADAMs and HB-EGF in sPLA_2_-IIA-induced EGFR transactivation. Although it is possible that other EGFR ligands could be also involved in sPLA_2_-IIA-induced EGFR transactivation, the fact that the presence of a HB-EGF-neutralizing Ab prevented the molecular and biological effects of the phospholipase suggests that HB-EGF plays a major role in the response induced by the sPLA_2_-IIA. We focused mainly on HB-EGF because of the extensive literature showing its role in cell survival and proliferation, both *in vivo* and *in vitro*[58-61]. Whether the remnant C-terminal fragment generated, HB-EGF-CTF, translocates to the nucleus and plays any role in sPLA_2_-IIA signaling should be investigated in greater detail in the future. Interestingly, transactivation of EGFR upon microglial stimulation with IFNγ also involves HB-EGF shedding, and is critical for the mitogenic and pro-inflammatory activity of this cytokine. This cross-talk mechanism between different signaling systems allows the integration of the great diversity of stimuli and supports the key role of the EGFR in diverse pathophysiological disorders.

Additionally, we showed that sPLA_2_-IIA induces rapid phosphorylation on Src at Tyr 416, and by using the selective inhibitor PP2 we demonstrated that Src participates in both HB-EGF shedding and EGFR phosphorylation at Tyr 845 and at Tyr 1173. Likewise, as already mentioned, EGFR phosphorylation at Tyr 845 (a Src phosphorylation site) is also diminished by MMP inhibitors, which indicates that products of MMPs are necessary for Src-mediated phosphorylation of EGFR at Tyr-845. Thus, it raises the possibility that EGFR ligands generated by MMP-mediated cleavage of membrane precursors collaborate with Src kinases in promoting sPLA_2_-IIA-induced EGFR transactivation. Therefore, our results suggest that Src contributes to sPLA_2_-IIA-induced EGFR transactivation at various steps: Src may serve as an upstream component of EGFR transactivation by phosphorylating Tyr 845 directly and indirectly by a MMPs/ADAMs/HB-EGF-dependent mechanism. These findings are consistent with abundant evidence indicating that external stimuli can transactivate EGFR in complex Src-dependent signaling [62-64]. Further studies are required to clarify the precise role of Src in this system, as well as to determine which member(s) of the family (Src, Fyn, and Yes) is involved in sPLA_2_-IIA-induced EGFR transactivation and BV-2 cells activation. It is possible that a particular member is involved in HB-EGF shedding and another one in EGFR phosphorylation at Tyr 845.

In contrast to Src signaling, sPLA_2_-IIA-activated MEK/ERK/MAPK and mTOR/P70S6K signaling pathways effectively seem to be downstream of EGFR transactivation. Thus, whereas the experimental conditions that affect HB-EGF release and EGFR phosphorylation abrogate phosphorylation of ERK, P70S6K and rS6, the presence of the specific inhibitors PD98059 (for MEK), or rapamicin (for mTOR) scarcely affects sPLA_2_-IIA-stimulated HB-EGF shedding and EGFR phosphorylation. In addition, our data suggest a complex, not linear, signaling network involving these two cascades, as the inhibition of any of those pathways prevents sPLA_2_-IIA-promoted activation of BV-2 microglia cells. It has been described that both pathways cross-talk extensively and may regulate each other both positively and negatively [65]. mTOR can be considered a key node of these complex signaling cascades, and exists as two different entities: the raptor-mTOR complex and the rictor-mTOR complex. Thus, it has been reported that phosporylation of P70S6K and its substrate, rS6, can take place in a rapamycin-dependent manner [66,67], or independently of mTOR, being Akt, ERK and even phosphatidic acid, direct upstream effector molecules [68,69]. Moreover, inhibition of the raptor-mTOR complex can trigger activation of the ERK/MAPK cascade, while inhibition of the rictor-mTOR complex inhibits Akt and ERK phosphorylation [70]. We have found that rapamycin, as well as PD98059, at concentrations that diminish or even suppress the proliferative and fagocytic capabilities of sPLA_2_-IIA-activated BV-2 cells, also suppress phosphorylation of ERK, P70S6K and rS6. In this study there was no attempt to investigate more deeply the effect of sPLA_2_-IIA on the sequential activation of these signaling proteins or the cross-talk between the raptor-mTOR/rictor-mTOR complexes. However, the relationship between these signaling pathways certainly deserves further, independent study due to the complex link existing between their components.

## Conclusions

In conclusion, our results reveal that sPLA_2_-IIA activates primary and immortalized BV-2 microglia cells; EGFR plays a key role as a critical regulator of this sPLA_2_-IIA-mediated effect, and also indicates that shedding of pro-HB-EGF is a crucial step in this response. Accordingly, the possibility that sPLA_2_-IIA may affect immune system function in the CNS in certain pathologies should be carefully considered.

## Abbreviations

Aβ: β-amyloid; ADAM: A disintegrin and metalloproteinase; AD: Alzheimer’s disease; CMK: Chloromethylketone; CNS: Central nervous system; COX-2: Cycloxygenase-2; DAPI: 4',6-diamidino-2-phenylindole; DMEM: Dulbecco’s modified Eagle’s medium; ELISA: Enzyme-linked immunosorbent assay; EGFR: Epidermal growth factor receptor; FCS: Fetal calf serum; FITC: Fluorescein-isothiocyanate; BB-EGF: Heparin-binding epidermal growth factor; hr-sPLA_2_-IIA: Secreted phospholipase A_2_-IIA human recombinant enzyme; IFNγ: Interferon-γ; IgG: Immunoglobulin; IL: Interleukin; LPS: Lipopolysaccharide; MMP: Metalloproteinase; OD: Optical density; PBS: Phosphate-buffered saline; PrI: Propidium iodine; PE: Phycoerythrin; pro-HB-EGF: Heparin-binding EGF-like growth factor; sPLA_2_-IIA: Secreted phospholipase A_2_-IIA; TNFα: Tumor necrosis factor-α; TAPI-1: TNFα proteinase inhibitor-1; TTBS: Tris-Tween buffered saline; TGF: Transforming growth factor.

## Competing interests

The authors declare that they have no competing interests.

## Authors’ contributions

RM and CC carried out the experiments and contributed to the manuscript. MLN designed the study, coordinated the experiments, analyzed data, prepared the figures, and contributed to the manuscript. All authors have read and approved the final version of the manuscript.
